# Renal and retroperitoneal metastasis from prostate adenocarcinoma: a case report

**DOI:** 10.1186/s12957-016-0834-4

**Published:** 2016-03-09

**Authors:** Chao Chen, Huadong He, Zhijian Yu, Yuansong Qiu, Xuliang Wang

**Affiliations:** Department of Urology, The First Hangzhou People’s Hospital, Huansha Road 261, Hangzhou, 310006 Zhejiang Province China

**Keywords:** Prostate adenocarcinoma, Metastasis

## Abstract

**Background:**

Diffuse renal and retroperitoneal metastasis of prostatic origin is an uncommon spread pattern of prostate cancer.

**Case presentation:**

We described a 74-year-old male patient who was admitted because of dysuria and nocturia. Physical examination and imaging study indicated prostate mass, and laboratory analysis revealed elevated prostate specific antigen (PSA). The diagnosis of prostate cancer was established after biopsy. In the further evaluation, diffuse renal and retroperitoneal metastasis of prostate cancer was confirmed. Radiotherapy combined with endocrine therapy was given.

**Conclusions:**

Our present case emphasized that the routine metastatic work-up was quite necessary, since a small proportion of men with advanced prostate cancer might experience metastases in atypical sites.

## Background

Renal and retroperitoneal metastasis from prostatic origin is an extremely rare clinical entity. Although prostate cancer has a recognizable pattern of spread, most often to regional lymph nodes and to the skeleton [1], many patients might present with atypical metastases at diagnosis [2]. We present a 74-year-old man who had a history of abnormally elevated prostate specific antigen (PSA) level. Radiological study suggested that the patient had diffuse retroperitoneal abnormal tissue and masses in the left kidney, which was confirmed to be metastasis from prostate cancer by biopsy. Renal and retroperitoneal metastasis from prostate cancer is a rare clinical entity. Necessary examination should be taken to make correct diagnosis.

## Case presentation

A 74-year-old male patient with 2-month history of dysuria and nocturia came to our hospital. Physical examination revealed mild edema of lower extremities. A hard nodule in the right lobe of prostate was detected in digit rectal exam. Laboratory data suggested an elevated PSA level of 36.801 μg/L. Prostate magnetic resonance imaging indicated that there was a solid mass in the right peripheral lobe of prostate with extracapsular extension and seminal vesicle invasion. The histological diagnosis of prostate cancer with Gleason score 5 + 4 = 9 was established by prostate biopsy.

**Fig. 1:**
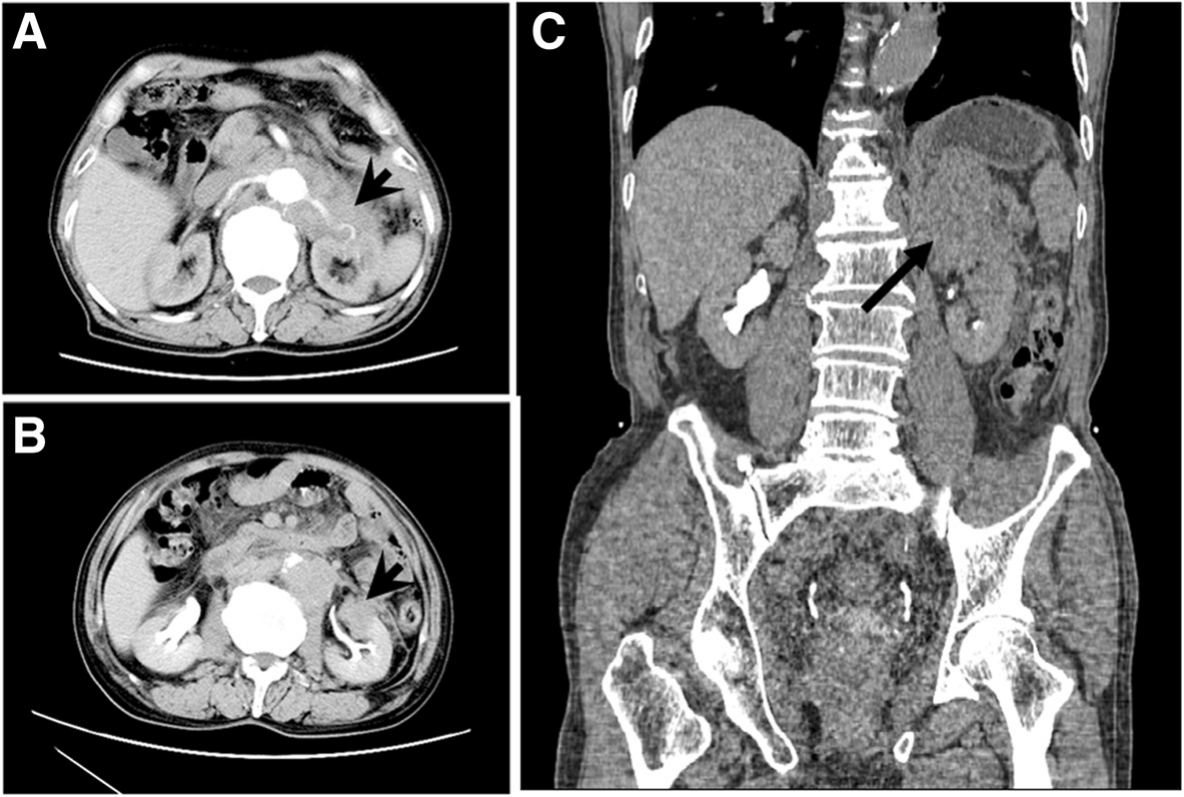
Computed tomography. **a** The arterial phase scanning image showing diffuse retroperitoneal mass wrapping the aorta and renal pedicle (*arrow head*), especially the left renal artery. **b** The excretory phase image showing the tumor (*arrow head*) located on the upper pole of the left kidney. **c** Coronal image revealed retroperitoneal mass with renal involvement (*arrow*)

**Fig. 2:**
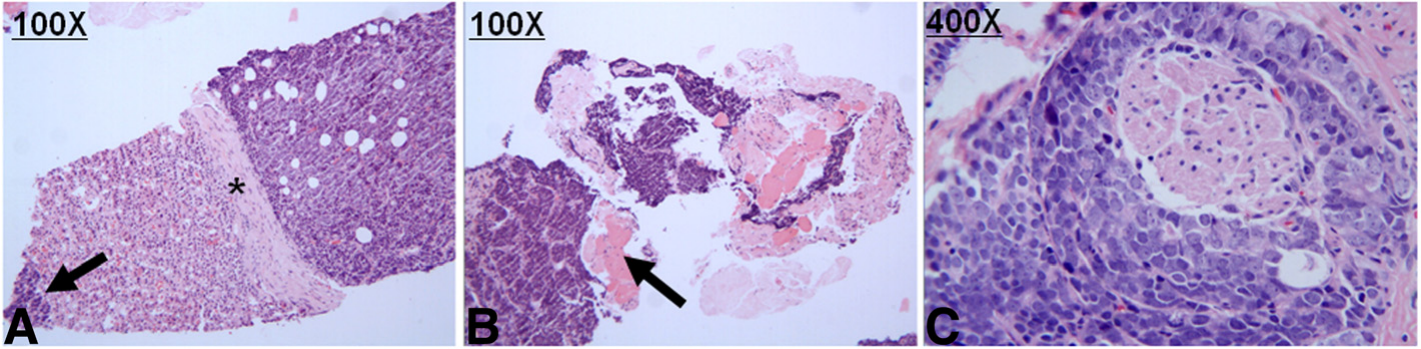
Histopathologic examination of renal and prostate biopsy. **a** Microscopic image revealed both renal metastases (*arrow*) and extra-capsular (*asterisk*) cancer tissue. **b** Microscopic appearance of retroperitoneal cancer with renal capsule (*arrow*) involvement. (H&E stain, ×100). **b** Retroperitoneal cancer with muscle (*arrow*) invasion. (H&E stain, ×100). **c** Prostate biopsy tissue (H&E stain, ×400)

In the further evaluation, skeleton scan was non-significant, but the ultrasonography found that there was a low echogenic mass in the upper pole of the left kidney. For further investigation, computed tomography (CT) was taken. The CT scan showed diffuse retroperitoneal low density mass wrapping the aorta and renal pedicle along with the left upper pole renal tumor (Fig. 1), which indicated the possibility of lymphoma. So the ultrasonography-guided retroperitoneal and renal mass biopsy was performed. The pathology (Fig. 2) suggested that the mass was adenocarcinoma, which was considered to be originating from the prostate according to the immunohistochemical stain. Considering the feature of metastatic prostate cancer, radiotherapy combined with endocrine therapy was given. However, the patient abandoned the treatment 1 month later because of economic reason and was lost in the follow-up.

### Discussion

Prostate cancer is the second most commonly diagnosed malignancy in men in the Western world and the second leading cause of cancer-related deaths among men worldwide [3]. Most prostate-cancer-related deaths result from distant metastasis [4]. The location of the metastatic lesion is not random, prostate cancer tends to spread to the skeleton [5]. Less frequently, it spreads to the liver, lung, bladder, or brain. Giorgio et al. [6] summarized 74,826 patients with metastatic prostate cancer from 1998 to 2010. They reported the most common metastatic sites were the bone (84 %), distant lymph nodes (10.6 %), liver (10.2 %), and thorax (9.1 %). Prostate cancer metastasis proceeds through a complex series of molecular events that include angiogenesis at the site of the original tumor, local migration within the primary site, intravasation into the blood stream, survival within the circulation, extravasation of the tumor cells to the target organ, and colonization of those cells within the new site [5].

Retroperitoneal involvement is an uncommon manifestation of prostate cancer. It is usually results from lymphatic spread of nodal metastases in ascending pattern. Metastases to the kidney are even more rare. To the best of our knowledge from literature searches, there are very few literatures that reported on renal metastasis from prostatic cancer. Philip et al. [7] reported a patient who was managed as a case of infected renal cyst which later turned out to be a metastatic prostatic adenocarcinoma with a rare pattern of widespread bony metastases. The authors suggested that suspicion was needed in order to avoid potential diagnostic pitfall. Fahd et al. [8] also reported a case with a left-sided renal mass found by radiological study. Subsequent histological examination and immunostain of biopsied tissue revealed feature characteristics of metastatic moderately differentiated to a focally poorly differentiated, large duct type of prostatic adenocarcinoma.

In our case, we also found the left kidney lesion incidentally. But unlike the previous two cases, the retroperitoneal space of our current patient was diffusely invaded by the metastatic lesion, which could possibly mislead to the diagnosis of lymphoma. Biopsy helped us establish the correct diagnosis. So we should bear in mind that although the skeleton is the most common site of prostate cancer metastasis, a small proportion of men with advanced prostate cancer might experience metastases in atypical sites. Thus, routine metastatic work-up was quite necessary.

## Conclusions

Metastasis work-up was quite necessary to rule-out extra-skeleton metastasis for those with high-risk prostate cancer, as a small proportion of patients with advanced disease would suffer from atypical site metastasis.

### Consent

Consent was obtained from the patient for publication of this case report.

## References

[1] Hess KR, Varadhachary GR, Taylor SH, Wei W, Raber MN, Lenzi R (2006). Metastatic patterns in adenocarcinoma. Cancer.

[2] Bubendorf L, Schopfer A, Wagner U, Sauter G, Moch H, Willi N (2000). Metastatic patterns of prostate cancer: an autopsy study of 1,589 patients. Hum Pathol.

[3] Siegel R, Naishadham D, Jemal A (2014). Cancer statistics, 2014. CA Cancer J Clin.

[4] Eccles SA, Welch DR (2007). Metastasis: recent discoveries and novel treatment strategies. Lancet.

[5] Hudson BD, Kulp KS, Loots GG (2013). Prostate cancer invasion and metastasis: insights from mining genomic data. Brief Funct Genomics.

[6] Gandaglia G, Abdollah F, Schiffmann J, Trudeau V, Shariat SF, Kim SP (2014). Distribution of metastatic sites in patients with prostate cancer: a population-based analysis. Prostate.

[7] Ibinaiye PO, Mbibu H, Shehu SM, David SO, Samaila MO (2012). Renal metastasis from prostate adenocarcinoma: a potential diagnostic pitfall. Ann Afr Med.

[8] Denti F, Wisard M, Guillou L, Francke ML, Leisinger HJ (1999). Renal metastasis from prostatic adenocarcinoma: a potential diagnostic pitfall. Urol Int.

